# Directional vibration sensing in the leafcutter ant *Atta sexdens*

**DOI:** 10.1242/bio.029587

**Published:** 2017-12-15

**Authors:** Felix A. Hager, Lea Kirchner, Wolfgang H. Kirchner

**Affiliations:** 1 Ruhr University Bochum, Biology and Biotechnology, 44780 Bochum, Germany; 2 Taita Taveta University, School of Agriculture Earth and Environmental Sciences, 80300 Voi, Kenya

**Keywords:** Substrate vibrations, Orientation, Directionality, Stridulation, Alarm signal

## Abstract

Leafcutter ants communicate with the substrate-borne component of the vibratory emission produced by stridulation. Stridulatory signals in the genus *Atta* have been described in different behavioural contexts, such as foraging, alarm signalling and collective nest building. Stridulatory vibrations are employed to recruit nestmates, which can localize the source of vibration, but there is little information about the underlying mechanisms. Our experiments reveal that time-of-arrival delays of the vibrational signals are used for tropotactic orientation in *Atta sexdens*. The detected time delays are in the same range as the time delays detected by termites. Chemical communication is also of great importance in foraging organization, and signals of different modalities may be combined in promoting the organization of collective foraging. Here we show that the tropotactic orientation to vibrational signals interacts with chemical communication signals.

## Abstract (precis)

**Summary:** Leafcutter ants communicate via substrate vibrations. Here we show that time delays between legs are used for orientation in a foraging context and that alarm pheromones interfere by changing the social context.

## INTRODUCTION

Substrate-borne vibrations play a crucial role in the communication in many insect groups (Cocroft and Rodríguez, 2005). Among others, they are used in the context of mate location, prey location and predator avoidance. To date, more than 50 behavioural studies demonstrate the ability of insects to localize the source of vibration, but there is little information about the underlying mechanisms (for reviews see Virant-Doberlet et al., 2006; Hill, 2009). In insects, vibration receptors are positioned in all six legs, which would be suitable for directional vibration sensing and vibrotropotactic orientation if time and/or amplitude differences are large enough to be processed in their central nervous system (Čokl et al., 2006; Virant-Doberlet et al., 2006). The only insect for which it has been demonstrated that the direction of a source of vibration on solid substrates can be perceived through simultaneous comparisons of the signal arriving at the legs is the termite *Macrotermes natalensis*. When termite soldiers are attacked by predators, they tend to drum with their heads against the substrate and create a pulsed vibration. Experiments with two movable platforms allowing vibration of the legs of the left and right sides of the body with a time delay, showed that the difference in time-of-arrival is the directional cue used for orientation. Delays as short as 0.2 ms are sufficient to be detected (Hager and Kirchner, 2014).

Ants should be confronted with a comparable selection pressure on the extraction of directional information out of the vibrational signal. In this context, leafcutter ants of the genus *Atta* are particularly interesting to study. The leafcutter ants can be reared in the laboratory and forage in the open. Workers can easily be motivated to enter certain experimental set-ups, simply by offering forage. *Atta sexdens* is relatively well studied in terms of both vibrational and chemical communication. For these and the following reasons, *Atta sexdens* is particularly suitable as a model system to investigate directional vibration sensing as well as its interplay with chemical communication signals. Like many other ant species, *A. sexdens* stridulates by raising and lowering their gaster, so that a cuticular file located on the first gastric tergite is rubbed against a scraper situated on the preceding third abdominal segment (Roces et al., 1993). *A. sexdens* and *Atta cephalotes* workers stridulate when they cut an attractive leaf. The stridulatory vibrations migrate along the body of the leafcutter ant and are transmitted from the ant's head to the substrate. Nearby workers respond to the stridulatory vibrations transmitted through the plant material by orienting towards the source of the vibration and subsequently join in leaf cutting (Roces et al., 1993; Roces and Hölldobler, 1996). Workers also stridulate when they are buried by a cave-in of the nest and thereby attract other workers, which subsequently start to dig and rescue the buried ant (Markl, 1967). Workers of *Atta vollenweideri* stridulate while engaged in nest digging and attract nestmates to join excavation activity at the same location, thus contributing to the spatial organization of collective nest building (Pielström and Roces, 2012). In all described social contexts, stridulatory signals are used to recruit nestmates. It is now clearly established, that leafcutters make use of vibrational signals to recruit nestmates. It would be beneficial for a recruit if directional information could be extracted out of the vibrational signal. We therefore hypothesized that recruits make use of time-of-arrival differences to localize stridulating nestmates. Our goal was to determine if workers of *A. sexdens*, straddling an experimental setup with two movable bridges, turn to the side vibrating first, and if so, what time-delays are sufficient for orientation.

Besides mechanical signals, communication in social insects is mediated primarily by chemical signals (Hölldobler and Wilson, 1990; Hunt and Richard, 2013). Often chemical and mechanical signals are intertwined in a multimodal communication system (Hölldobler, 1999). In social insect societies, where individuals frequently live in large and complex colonies, the constant exchange of information through an array of sophisticated social behaviours acts to synchronize interactions between individuals, thereby promoting efficient foraging, reproduction and defence (Buczkowski and Silverman, 2005). Chemical signals play a fundamental role in information transfer between and among individuals, nestmate recognition, colony cohesion, behaviour, and task regulation (Richard and Hunt, 2013). Chemical signals can inform colony members of the presence of danger. In *A. sexdens* the mandibular gland secretions comprise a complex mixture of volatiles that elicit alarm behaviour (Francelino et al., 2006). Typically observed alarm behaviour in *A. sexdens* includes increased antennation, vigorous movement and opening the mandibles (Francelino et al., 2006). The context in which a receiver perceives a signal is of crucial importance and can affect the behavioural response (Hölldobler, 1999). Chemical communication is of great importance in ant foraging organization and combined signals of different modalities promote the organization of collective foraging (Jackson and Ratnieks, 2006). We therefore confronted workers simultaneously with a vibratory stimulus and the pheromone component citral. Citral is a monoterpene present in the mandibular gland secretion of the leafcutter ant *A. sexdens* that repels workers (Blum et al., 1968; Roces, 1994; Francelino et al., 2006). We hypothesized that workers show a positive tropotaxis to vibrational stridulation signals in a foraging context, and that the cue they use for orientation is a time delay of the arriving vibrational wave at different legs. By confronting the foragers with an alarm pheromone, the context in which the vibrational stridulation signals are perceived is changed. Alarm signals should either supersede recruiting signals or intertwine to multimodal signals. We therefore hypothesize that ants do not show a side preference to vibrational stridulation signals when confronted simultaneously with an alarm pheromone.

## RESULTS AND DISCUSSION

**Fig. 1.**
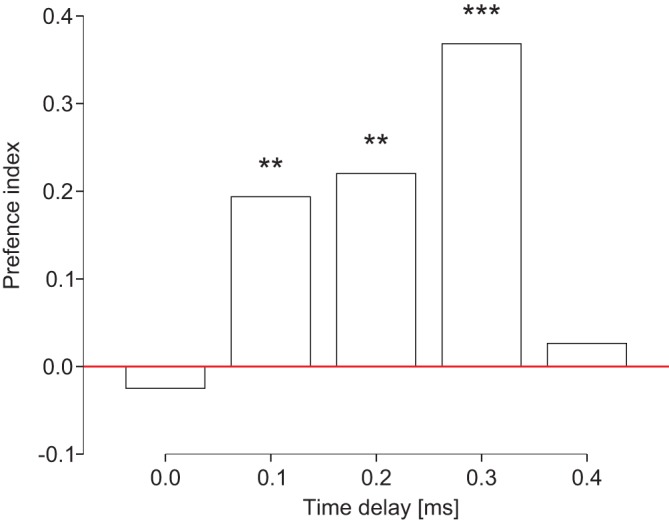
**Preference indices of *A. sexdens* in relation to the time delay of the vibrational stimuli generated with two movable bridges.** In the control experiment with both bridges vibrating at the same time, ants do not show a side preference (*P*=n.s.; *n*=80). When the bridges are vibrated with time delays of 0.1 ms (*P*<0.002; *n*=253), 0.2 ms (*P*<0.003; *n*=177) and 0.3 ms (*P*<0.001, *n*=76) ants turn significantly more often to the side vibrating first. Asterisks indicate significant side preferences (Chi²-analysis; **P<0.01, ***P<0.001).

Ants standing with the legs of one body side on a vibrating L-bridge and with the legs of the other body side on a L-bridge vibrating a short moment later turn towards the side that vibrates first (Fig. 1). In the control experiment with both sides vibrating at the same time, ants do not show a side preference (*P*=n.s.; *n*=80). We tested four time delays. In experiments with time delays of 0.1 ms (*P*<0.01; *n*=253), 0.2 ms (*P*<0.01; *n*=177) and 0.3 ms (*P*<0.001; *n*=76), ants turn significantly more often to the side vibrating first. If the sides vibrate with a time delay of 0.4 ms (*P*=n.s.; *n*=76) the effect cannot be supported statistically. Our results confirm indirect evidence from behavioural studies in various insects (Cocroft et al., 2000; Virant-Doberlet et al., 2006; Hill, 2009; Roces et al., 1993). They also demonstrate that the body size of many insects is sufficient for a vibrotropotactic orientation based on the analysis of time-of-arrival delays. Directional information is thus available in the analysis of time-of-arrival delays between legs positioned on the substrate. Foraging leafcutters stridulate while cutting leaves. Vibrational signals are transmitted in plant stems as bending waves, for which the transmission velocity increases with the square root of frequency (Michelsen et al., 1982). As a result, any signal with multiple frequencies will become increasingly distorted over distance (Cocroft et al., 2013; Cocroft and Rodríguez, 2005). Very few studies have been conducted regarding the transmission of vibrational waves in plant stems. Michelsen et al. (1982) found that the amplitude of vibrational waves travelling through plant stems varies greatly and does not simply correlate with the distance from the source. The utilization of amplitude differences for orientation by ants therefore seems less likely than the utilization of time delays. Most energy of stridulatory vibrations is found between 1 and 3 kHz (Markl, 1968). We did not measure the velocity of bending waves travelling in *A. sexdens* foraging trees. Rush stems (*Juncus effusus*) can transmit dispersive and non-dispersive bending waves, and wave velocity is proportional to the square root of the frequency at low frequencies (Casas et al., 2007). Measurements of other plant species suggest that the velocity of a bending wave could be roughly in the range of 120–220 ms^−1^ (Michelsen et al., 1982). The distance between the vibration receptors of the ants' legs is crucial to assess whether this velocity leads to time delays which could be processed. The caste system of leafcutter ants of the genus *Atta* are among the most complex in social insects, involving extreme size variations (Hölldobler and Wilson, 1990). The distance between the front leg of one body side and the hind leg of the other body side is approximately 20 mm, leading to time delays of approximately 0.09–0.17 ms between legs. The temporal resolution abilities of the ants, therefore, seems to be sufficient to solve the task of directional orientation. Very small time delays close to zero and very large time delays in the range of 0.4 ms measured in a distance similar to that between the ant's legs positioned on the substrate do not occur in natural substrates. Therefore, it is not surprising that ants do not show a side preference. On a physiological level, the temporal resolution of vibrational direction sensing has been studied in *Locusta migratoria* (Čokl et al., 1985). The response pattern of ventral cord neurons depends on the direction and the time delay of the presented vibrational stimulus. Directional processing occurs at the ventral cord level by integrating the inputs from the vibratory receptors from several legs. Because of the locust's relatively large size, receptors in different legs are approximately 5 cm apart, leading to a time delay between 0.4 and 4 ms (Čokl et al., 1985). Leafcutter ants routinely move through different environments while foraging and are found on diverse surfaces such as the nest substrate, the surrounding soil, plant stems and leaves. The transmission properties may dramatically differ from substrate to substrate. In this context, it would be interesting to analyse the vibrations produced by signalling ants and to answer the question, whether leafcutter ants pursue a generalist strategy, by producing signals that are effective across the range of substrates they typically encounter.

**Fig. 2.**
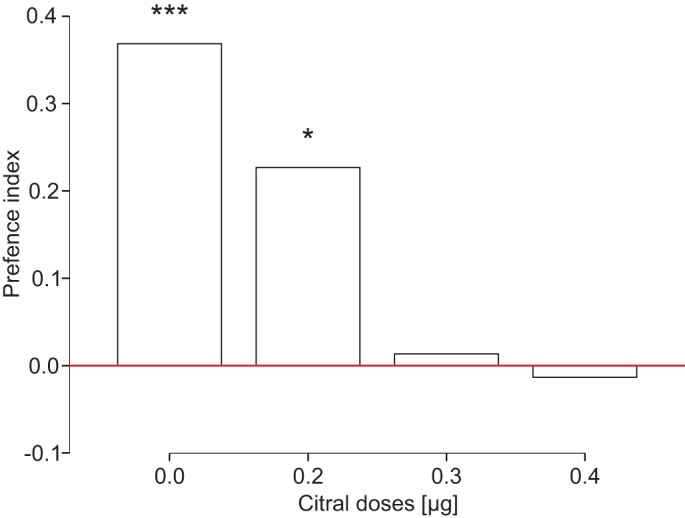
**Preference indices of *A. sexdens* in relation to a time delay of 0.3 ms of the vibrational stimuli generated with two movable bridges and different citral doses.** Without the presence of citral (*P*<0.001, *n*=76) and with a low dose of citral (0.2 μg citral; *P*<0.05; *n*=75) ants turn significantly more often to the side vibrating first. In the presence of higher citral doses of 0.4 μg citral (*P*=n.s.; *n*=75) and 0.6 μg (*P*=n.s.; *n*=75) the ants show no side preference. Asterisks indicate significant side preferences (Chi²-analysis: *P<0.05; **P<0.01; ***P<0.001).

Furthermore, the directional response of the ants is modulated by citral. The presence of citral, a compound of the ants' alarm pheromone, modulates their directional response to vibrational stimuli significantly (Fig. 2). We tested with a time delay of 0.3 ms. In the control experiment without citral (*P*<0.001; *n*=76) and in the presence of a low dose of 2 μg citral (*P*<0.05; *n*=75) the ants turn towards the side that vibrates first. Confronted with higher doses of 0.4 μg (*P*=n.s.; *n*=75) and 0.6 μg (*P*=n.s.; *n*=75) citral the ants show no side preference. The ants seem to pay less attention to directional cues leading them to profitable food sources when they are confronted with a chemical alarm signal and a recruitment signal at the same time. This supports the idea that vibrational signals constitute an important and long underestimated part in recruitment communication and interact with chemical signals in multimodal communication.

## MATERIAL AND METHODS

Experiments were conducted with a queenright laboratory colony of *A. sexdens* (kept since 2009), from October 2014 to June 2015 at the Ruhr University Bochum, Germany. Food, i.e. fresh leaves of *Rubus* sp., was provided at the foraging site in the morning. Experiments started as soon as the ants frequently used a bridge to access the foraging site. Per day each ant was tested only once. At the end of each day tested ants were returned to the colony. Due to the high number of individuals in the colony (approximately 10,000) we considered the possibility to test individuals twice very unlikely and therefore treated our measurements as independent.

**Fig. 3.**
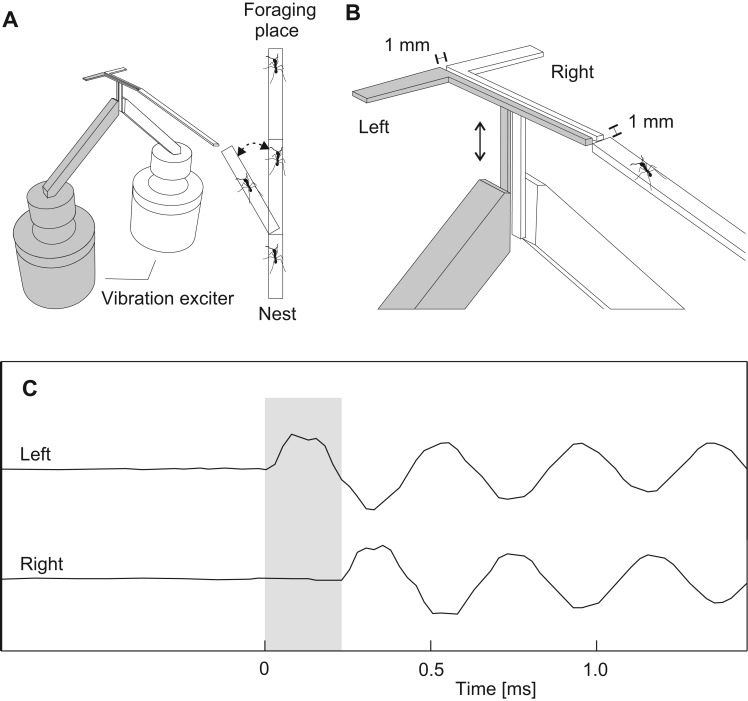
**Experimental setup.** (A) Foraging *A. sexdens* walked over a bridge and entered the experimental setup. (B) Two L-bridges were vibrated independently with short time delays when an ant walked with three legs on one side and with the three other legs on the other side. Arrow indicates direction of vibration. (C) Oscillogram of a sinusoidal 2.7-kHz artificially generated stridulatory signal record simultaneously on the L-bridge vibrating first (left) and on the L-bridge vibrating 0.2 ms later (right). Grey bar indicates 0.2 ms time delay.

The detection of time delays was studied using artificial stridulation signals induced by a function generator (Rigol, Beaverton, USA, DG 1032). Artificial stridulation signals were 2.7 kHz sinusoidal pulses with a pulse duration of 33 ms and a pulse repetition rate of 4.5 Hz. These temporal characteristics resemble the natural stridulating signals of the genus *Atta* (Markl, 1968). An experimental setup in which the legs of both body sides could be vibrated independently was adapted for *A. sexdens* (Fig. 3A). Leafcutter ants employ stridulation signals in different social contexts. We wanted to test large workers in a foraging context. Therefore, we tested ants that walked on the bridge into the direction of the foraging place. A second small bridge was briefly connected with one end to the bridge connecting the nest with the foraging arena and with the other end to two L-bridges, allowing foragers to enter the experimental setup. The L-bridges were constructed with a very light and stiff glass-fibre-reinforced epoxy (long side 45 mm, short side 21 mm, width of pathway 2 mm, material thickness 1 mm) to ensure suitable vibration transmission. A gap of 1 mm width between the L-bridges and the connecting bridge ensured that only the L-bridges were vibrated (Fig. 3B). The connecting bridge was removed after a worker entered the L-bridges. The L-bridges were vibrated as soon as the ant entered. Both L-bridges were vibrated in exactly the same way, except for a short time delay (Fig. 3C). The L-bridge that vibrated first was chosen randomly. A gap of 1 mm width between the L-bridges ensured mechanical isolation. The amplitude of the vibrational signal was calibrated to 1 ms^–2^ peak to peak measured under the mounting point. This is in the range of natural stridulation signals. Stridulating *A. cephalotes*, for example, induce peak to peak amplitudes on leaves of over 10 ms^−2^ measured in 2 cm distance (Tautz et al., 1995). The amplitudes of the stimuli were controlled using two accelerometers (B&K 4381) permanently mounted under the bridges, two charge amplifiers (B&K, Nærum, Denmark, 2635) and an oscilloscope (Tektronix, Beaverton, USA, TBS 1022). Additionally, measurements at different points on the two bridges were conducted with a laser doppler vibrometer (Polytec, Waldbronn, Germany, PDV 100) to ensure that both bridges vibrate in the same way. After crossing the straight part of the L-bridges, ants had to decide to turn to the right or the left side. Ants entering the experimental setup were videotaped. The video analysis allowed us to take only ants into account which walked over the L-bridges with three legs on one side and the other three legs on the other side. The video analysis was done by an observer, who was not aware which side vibrated first. It was regarded as a reaction towards the source of the vibration when the ant turned to the side that vibrated first. Preference indices (xi) were calculated: xi=a–b/a+b; with a=ants turning to the side vibrating first and b=ants turning to the side vibrating later. In addition to time delays of 0.3 ms we confronted ants with different doses of citral. Therefore, a piece of filter paper with the different citral doses were placed under the L-bridges.
